# Human intuition as a defense against attribute inference

**DOI:** 10.1038/s41598-023-43062-5

**Published:** 2023-09-26

**Authors:** Marcin Waniek, Navya Suri, Abdullah Zameek, Bedoor AlShebli, Talal Rahwan

**Affiliations:** 1 Computer Science, Science Division, New York University Abu Dhabi, Abu Dhabi, UAE; 2 Social Science Division, New York University Abu Dhabi, Abu Dhabi, UAE

**Keywords:** Computational science, Computer science

## Abstract

Attribute inference—the process of analyzing publicly available data in order to uncover hidden information—has become a major threat to privacy, given the recent technological leap in machine learning. One way to tackle this threat is to strategically modify one’s publicly available data in order to keep one’s private information hidden from attribute inference. We evaluate people’s ability to perform this task, and compare it against algorithms designed for this purpose. We focus on three attributes: the gender of the author of a piece of text, the country in which a set of photos was taken, and the link missing from a social network. For each of these attributes, we find that people’s effectiveness is inferior to that of AI, especially when it comes to hiding the attribute in question. Moreover, when people are asked to modify the publicly available information in order to hide these attributes, they are less likely to make high-impact modifications compared to AI. This suggests that people are unable to recognize the aspects of the data that are critical to an inference algorithm. Taken together, our findings highlight the limitations of relying on human intuition to protect privacy in the age of AI, and emphasize the need for algorithmic support to protect private information from attribute inference.

## Introduction

In recent years, the algorithms that predict our attributes based on publicly available information have reached staggering levels of effectiveness and sophistication. Easy access to vast amounts of high-resolution data has granted AI algorithms almost clairvoyant-like powers. By analyzing our digital footprint an algorithm can judge our personality traits better than our loved ones^1^, by processing a photo of our face it can uncover our sexual orientation^2^, and by scrutinizing the dynamics of our keystrokes it can infer our emotional state^3^. While incredibly impressive as a technological achievement, many consider these advancements in prediction techniques deeply unsettling, since the attributes that can easily be inferred by such techniques include sensitive data that can be used against us. For instance, knowledge about personality traits and emotional states can be used to manipulate one’s behavior^4^, while knowledge about sexual orientation can lead to discrimination in certain parts of the world. Mass applications of AI-driven surveillance technologies by authoritarian regimes can significantly strengthen their control over the population^5^. Many fear that living under the ever-watchful eye of artificial intelligence will lead to a new kind of technological dystopia^6^.

Many of the privacy solutions in the literature are based on the role of a centralized authority. Notions such as *k*-anonimity^7^, differential privacy^8^, and federated learning^9^ work towards securing sensitive information under the assumption that a set of rules will be provided and obeyed by a central governing force. However, real-life institutions can be prone to error, negligence, or even malice, as evidenced by numerous privacy-related scandals in the recent years^10^. One possible solution to this issue would be to put responsibility for privacy protection into the hands of the general public, letting them strategically shape their publicly available data in order to guard the information they deem sensitive.

In this work, we examine the feasibility of people protecting their own privacy from attribute inference, without any kind of algorithmic help. In particular, we consider two main research questions. First, *how effective are people in inferring private attributes, when compared to algorithms?* While privacy protection remains the primary motivation behind our study, understanding people’s ability to infer hidden information could help us understand how they think when attempting to hide such information. Second, *how effective are people in hiding private attributes from inference, when compared to algorithms?* If members of the general public are able to conceal information from AI without any algorithmic support, then it would be enough to simply inform them about the potential risk of their sensitive data being uncovered. If, on the other hand, they are incapable of hiding private attributes on their own, it would underscore the need for developing algorithms that can support them in this endeavor.

In more detail, we consider three different attributes that can be inferred based on publicly available data: the gender of the author of a review, the country in which a set of photos was taken, and a missing link in a social network. For each of these attributes, we compare the performance of participants against AI algorithms in two tasks: inferring the attribute, as well as preventing its inference (i.e., modifying the publicly available data in order to make it harder for algorithms to infer the private attribute). Our results help us understand people’s ability to take the safety of their sensitive information into their own hands.

## Results

**Figure 1:**
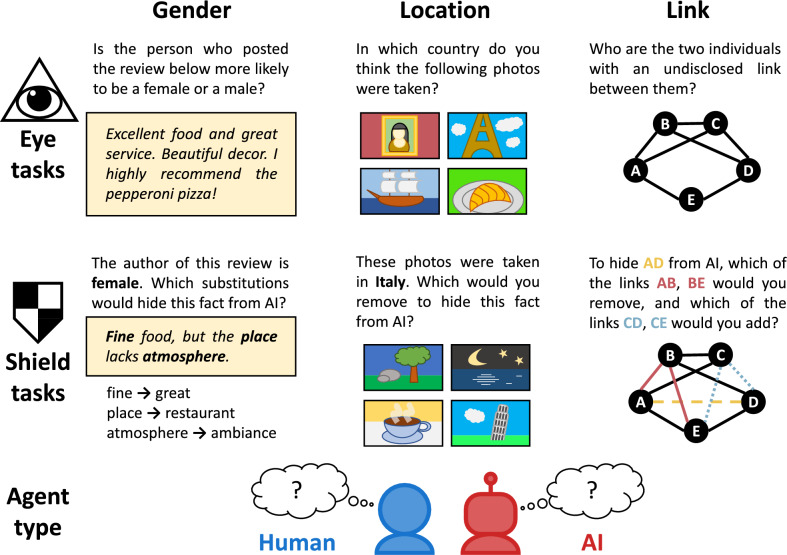
The general outline of our experiment. We focus on three attributes: (i) the *gender* of the author based on the text of the review, (ii) the *location* of origin based on a set of pictures, and (iii) the undisclosed *link* based on the structure of a social network. For each of these attributes, we consider the *eye task*, which involves inferring the attribute based on available data, and the *shield task*, which involves modifying the data in order to make it harder for an AI algorithm to infer the attribute. Each of the six tasks is given to two types of agents: people (participants recruited online), and AI (algorithms trained on data), in order to compare their performance.

Our analysis focuses on three attributes: (i) the *gender* of the author of a particular piece of text, (ii) the *location* in which a particular set of photos was taken, and (iii) the undisclosed *link* in a particular social network. For each of these attributes, we focus on two distinct tasks, and refer to the entity solving these tasks as an *agent*, which could either be a human or an algorithm. In the first task, the attribute of interest is hidden, and the agent is required to infer this attribute from the given data. For example, in the case of gender, the agent is presented with a piece of text, and is required to infer the author’s gender. In the second task, the attribute of interest is revealed, and the agent is required to modify the given data in order to make it harder for an algorithm to infer that attribute. For example, in the case of gender, the agent is presented with a piece of text along with the author’s gender, and is asked to modify the text in order to keep author’s gender hidden from prediction algorithms. The first task will be referred to as the *eye task*, since involves “seeing” hidden information, while the second task will be referred to as the *shield task*, since it involves “protecting” hidden information. The general outline of all eye and shield tasks is illustrated in Fig. 1.

We now briefly comment on generating the instances of the tasks. The technical details of the process are presented in the Methods section. For the gender attribute, we generate the corresponding task instances using a dataset of Yelp reviews^11^. Each eye instance consists of the text of a review. To construct a shield instance, we select four words of the review that are most indicative of the author’s gender, as well as four words that are least indicative. We measure how indicative a given word is according to the normalized pointwise mutual information^12^, the criterion employed by Reddy and Knight^11^. The agent is then asked to select three of these eight words to be substituted by their synonyms. For the location attribute, we use a dataset of Flickr photos^13^. Each eye instance consists of a set of 16 randomly chosen photos taken in the same country. To construct a shield instance, we identify four photos whose removal yields the largest drop in the location prediction accuracy, as well as four photos whose removal yields the smallest drop. The agent is then asked to select three of these eight photos to be removed. For the link attribute, we generate networks using three different models: Barabási-Albert ^14^, Erdős-Rényi ^15^, and Watts-Strogatz ^16^. Each eye instance consists of a network from which we randomly remove one link (this is the undisclosed link that the agent is asked to identify). To construct a shield instance, we identify two links whose removal causes the greatest decrease in the effectiveness of link prediction, as well as two links whose removal causes the least decrease. Moreover, we identify two links whose addition causes the greatest decrease, as well as two whose addition causes the least decrease. The agent is then asked to select three of these eight modifications to be introduced.

We recruited participants using Amazon Mechanical Turk^17^. The online questionnaire is presented in Section A of the Supplementary Materials. Altogether, 1168 participants solved the comprehension test and completed their tasks. The exact distribution of participants per task is presented in Table S1 in the Supplementary Materials. The participants’ number was determined using a power analysis of the pilot study. We preregistered our main findings using the AsPredicted.org portal^18^.

We now briefly comment on the information given to the participants. The task is explained to them, and their comprehension is tested, but they are not being trained in the task beforehand. This is unlike the machine learning algorithms, which were trained using the training set. We made this design decision since we are primarily interested in evaluating the potential use of human intuition as a privacy defense tool, rather than evaluating the ability of participants to be rigorously trained in these tasks. To put it differently, we are interested in understanding the performance of an average Internet user, without any specialized guidance. Nevertheless, an interesting idea for a future study would be to investigate people’s learning curve when it comes to privacy protection tasks.

**Figure 2:**
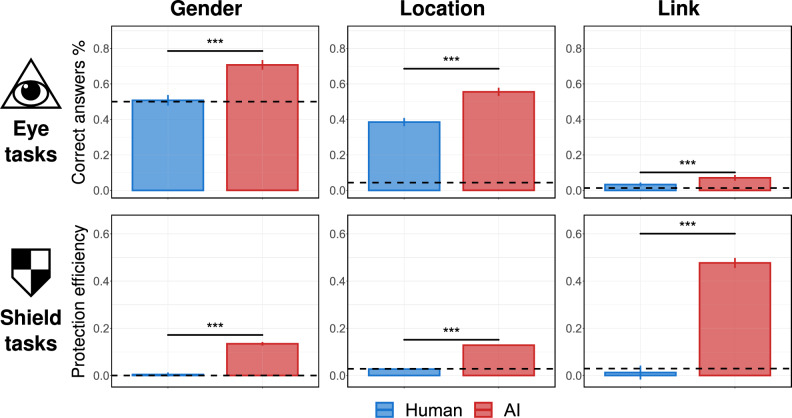
Average performance of people vs. algorithms in the eye tasks and the shield tasks. Each column corresponds to a different attribute (gender, location, and link). The first row presents results of the eye tasks with *y*-axes corresponding to the percentage of the correct answers. The second row presents results of the shield tasks, with *y*-axes corresponding to the protection efficiency (see Methods). Each plot compares the average performance of survey participants and AI algorithms in a given task, with the dashed line highlighting the performance of a random baseline. Error bars represent $95\%$ confidence intervals. All results are significant with *p*-values smaller than 0.001 according to the Welch’s *t*-test (the exact values are presented in Table S1 in the Supplementary Materials).

**Figure 3:**
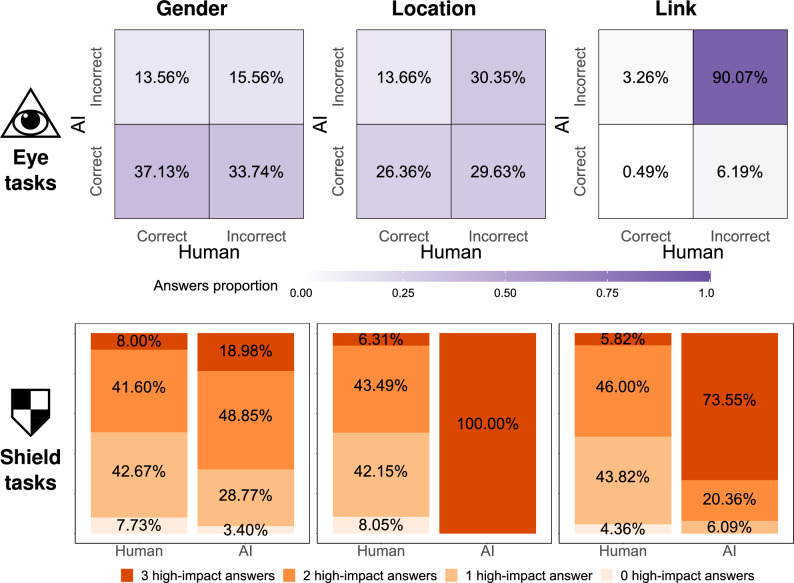
Comparison of correct/incorrect and high-impact/low-impact answers in the eye tasks and the shield tasks. For any given eye instance, the algorithm assigns probabilities to different answers, and is evaluated based on the one with the highest likelihood of being correct. To provide a similar evaluation to humans, they are evaluated based on the most common answer. The first row focuses on the eye task, comparing the percentage of instances that were correctly and incorrectly answered by the participants and by AI. The second row focuses on the shield task, comparing the percentage of high-impact and low-impact answers selected by the participants and by the AI.

Figure 2 compares the performance of participants and AI algorithms in the eye tasks and the shield tasks. As can be seen, AI outperforms humans in every task. When we focus on the eye tasks, the average performance of both types of agents is the greatest in gender prediction, followed by location prediction, with the average performance in link prediction being the poorest. This is consistent with the number of possible answers to each eye instance, as the agent has to choose one of two genders, one of twenty three countries, or one of about seventy five possible links, respectively. When we turn our attention to the shields tasks, the average performance of AI is inversely proportional to the performance in the corresponding eye task. In other words, if the problem is already difficult even without any strategic obfuscation, it makes it easier to add an additional layer of confusion. However, the situation is much more dire for the human agents attempting to perform the shield task, as their average performance seems to be comparable to the random baseline. Altogether, our results present a rather dire perspective of human ability compared to AI. Not only are humans consistently outperformed by algorithms throughout all settings, but their ability is particularly lacking when it comes to privacy protection (i.e., the shield task). These findings underscore the need for both systemic solutions that guard our sensitive information, as well as tools and techniques that would assist us in taking the responsibility for protecting our privacy in our own hands.

Figure 3 gives us a deeper insight into the differences in performance between humans and AI. For about half the instances of the gender- and location-based eye tasks, the answers of human and AI agents are either both correct, or both incorrect. The gap in performance results from the distribution of the other half. More specifically, the number of instances in which AI, but not humans, give a correct answer is two to three times greater than those in which humans, but not AI, give a correct answer ($33.74\%$ vs. $13.56\%$ for gender, and $29.63\%$ vs. $13.66\%$ for location). This difference is the source of the AI’s competitive advantage. As for the link attribute, we observe a much greater percentage of instance where both types of agents give incorrect answers ($90.07\%$). Again, when it comes to the instances in which the outcomes differ, we see about twofold difference in AI’s favor ($6.19\%$ vs. $3.26\%$). We now turn our attention to the shield tasks. Notice that for each of the three attributes, every instance has four of the possible modifications selected as having the greatest impact on prediction quality (we refer to these as the *high-impact* answers), and the other four selected as having the smallest impact (we refer to these as the *low-impact* answers). The figure shows that when trying to hide private information, people consistently select a smaller percentage of high-impact answers than AI. The difference is especially pronounced in the case of location, as the inference algorithm simply takes a linear combination of the scores of all photos. Thus, an AI algorithm trying to obscure the location information has a relatively easy task of minimizing the accuracy. However, even for the other two attributes, where the prediction algorithms are non-linear, the ability of AI agents to identify high-impact answers is much greater than human agents. This finding suggests that the inability of people to discern what aspects of the given instance are meaningful to the AI may be responsible for their poor performance—a conclusion that is consistent with previous findings from the literature^19^.

## Discussion

In this work, we compared the ability of human and AI agents to infer private information based on publicly available data, as well as their ability to protect such information against inference attacks. We found that the performance of people is significantly inferior to that of algorithms in both tasks, across all considered attributes. This deficiency of human capability is particularly worrying when protecting one’s privacy, as the performance of our study participants is close to the random baseline. To better understand the reasons behind this performance gap between humans and machines, we took a closer look at the agents’ answers. When considering the problem instances that were correctly solved by only one of the two agent types, the AI solved two to three times more instances than people. Moreover, when it comes to private data protection, the participants were much less likely to use data modifications that have high impact on prediction accuracy. While the general trends of our results might be expected, given the phenomenal ability of machine learning models to identify correlation in data, our work quantifies the AI’s advantage in measurable terms, and our findings highlight the need to use algorithms to protect people’s privacy in the age of AI.

Most of the works related to privacy protection focus on the role of a centralized authority in preserving the safety of information. Common notions of privacy include *k-anonimity*^7^, which guarantees that any individual is indistinguishable from at least $k-1$ others, *differential privacy*^8^, which guarantees that based on the output of an algorithm it is impossible to determine whether the data of a given individual was part of the input, and *federated learning*^9^, where data is spread over multiple entities, and no one has access to the complete information. However, all these methods put the responsibility for protecting the private information of individuals in the hands of a central authority, which might be prone to error and negligence. In contrast, our work tests the ability of people to protect their own privacy. Another relevant body of literature concerns the leakage of private information from the increasingly popular large language models^20–22^. In particular, research shows that when correctly prompted, language models can be forced to disclose personally identifiable information existing in the training data, despite the use of various scrubbing techniques. In comparison, our work concerns itself with the inference of private information in the test data, rather than the disclosure of information from the training data.

The part of our study concerning gender and location prediction is closely related to the field of adversarial machine learning^23^, which considers the process of doctoring inputs of machine learning algorithms. In particular, our study can be classified as part of the literature on evasion attacks^24^, where adversarial modifications are introduced in testing instances, as opposed to poisoning attacks^25^, where the training data gets altered. However, most of the literature considers the situation where the data is modified by algorithms, whereas we put this task in the hands of people. A recent work evaluated the capability of social media users to understand what features of one’s Twitter activity are most revealing to algorithms predicting our opinions^19^. However, the authors did not test how modifying said features affects the accuracy of prediction, whereas we perform this evaluation in all settings that we consider.

The part of our study concerning the link prediction tasks is also related to the growing literature on strategically obscuring information from social network analysis tools. Some works proposed heuristic strategies of hiding certain relationships from link prediction algorithms based on the knowledge about local network neighborhood^26, 27^. A similar problem was considered for evading sign prediction algorithms, whose primary goal is to predict whether a particular link is positive or negative in a given social network^28^. Other works have considered evading a variety of network analysis tools, including centrality measures^29–35^, community detection algorithms^36^, and source detection algorithms^37^. Compared to those works, the novelty of our approach lies in the examination of people’s ability to infer private information, their ability to protect such information from inference attacks, and how their ability compares to that of algorithms designed specifically for this purpose.

We now discuss the policy implications of our results. The observed inadequacy of people to effectively protect private information from being inferred by AI underscores the need for new solutions to assist humans in these tasks. Otherwise, people may rely on their intuition, introducing certain modifications to their data before sharing it publicly, with the false belief that such modifications will safeguard them against attribute-inference attacks. As we have demonstrated, using algorithms is much more reliable than using one’s intuition. This highlights the need to develop algorithms that can modify people’s data, allowing them to share it with others, while ensuring that their secrets cannot be inferred from the shared content. Although people could outsource the task of data protection to a central authority, e.g., the company behind the social media platform they use, such an approach may be ineffective, as indicated by a plethora of privacy-related scandals. As such, users of the World Wide Web require tools and techniques that they can apply themselves to safeguard the information that they deem sensitive. Such tools might take the form of automated assistants^38^, simple rule-of-thumb rules based on the inner working of the prediction algorithms^27, 36^, or entire applications devoted to the task of privacy preservation^39, 40^.

## Methods

### Generating gender prediction instances

To generate the gender instances, we use a dataset of about 81, 000 reviews left by the users of Yelp^11^. Each review is labeled as posted by either a male or a female. We use the dataset to train an L2-regularized logistic regression classifier with bag-of-words count, following the work of Reddy and Knight^11^, using an 80 : 20 train-test split. To generate the set of eye task instances, we select 1000 reviews uniformly at random from the test set, and participants are asked to specify whether the review was written by a male or by a female.

We also generate 1000 shield task instances based on reviews selected uniformly at random out of all reviews in the test set. We select four words of the review that are most indicative of the correct label, and four that are least indicative (according to the normalized pointwise mutual information^12^). We allow the substitution of each of these eight words for its closest semantic equivalent computed using the *word2vec* extension by Levy and Goldberg^41^. Participants are then asked to select three out of eight possible substitutions. To discern the participants’ ability to solve the shield task, we ensure that the instances used in our experiments have a pool of answers that vary in terms of solution quality. More formally, in terms of the probability assigned to the correct gender by the prediction algorithm, we ensure that the possible solutions (i.e., the possible sets of three substitutions) include solutions that increase this probability by at least 0.025, and also include solutions that decrease this probability by at least 0.025. Moreover, we ensure that the standard deviation of the quality of possible solutions (i.e., the changes in the aforementioned probability) is at least 0.025. The process of randomly selecting instances is repeated until all instances satisfy the above conditions.

### Generating location prediction instances

To generate the location instances, we use a dataset of about 750, 000 photos downloaded from Flickr, provided by Yang et al.^13^. Each photo is labeled with the name of the country in which it was taken, selected from the following: Australia, Cambodia, Canada, China, Cuba, France, Germany, India, Ireland, Italy, Japan, Mexico, Netherlands, New Zealand, Norway, Peru, Portugal, Spain, Switzerland, Taiwan, Thailand, United Kingdom, and United States of America. Using this dataset, we train a deep convolutional neural network to produce, for any given photo, a probability distribution over all countries. To this end, we apply a standard VGG-16 architecture^42^, using an 80 : 20 train-test split. Following Yang et al.^13^, the probability distribution for any given set of photos is generated as a product of the probability distributions corresponding to the photos in that set. Following Yang et al.^13^, each eye task instance consists of a set of 16 photos taken in the same country, selected uniformly at random out of all photos taken in that country. When generating the instances, we ensure that they have the same distribution of countries as the original dataset; we ended up with 1010 instances. Notice that we select the photos belonging to the instances from the test set. Participants were then asked to select the country in which the photos were taken.

As for the shield task instances, we generate the same number of instances as the eye task instances (i.e., 1010) with the same number of photos per instance (i.e., 16) selected from the test set, while ensuring that they have the same distribution of countries as the original dataset. Participants must select three photos to be removed from the set, with the goal being to hide the country in which the photos were taken, i.e., to minimize the probability assigned by the classifier to the correct country. To narrow down the possible photos that the participants can choose from, we allow them to choose from only eight photos, consisting of four photos whose removal results in the greatest drop in probability, as well as four photos whose removal results in the smallest drop. Again, to discern the participants’ ability to solve the shield task, we ensure that the instances used in our experiments have a pool of answers that vary in terms of solution quality. That is, we ensure that the possible solutions include one that increases the probability by at least 0.025, and another that decreases the probability by at least 0.025, while also ensuring that the standard deviation of changes in probability is at least 0.025.

### Generating link prediction instances

To generate the networks that are part of the link prediction instances, we use three models, namely Barabási-Albert ^14^, Erdős-Rényi ^15^, and Watts-Strogatz ^16^. We generate networks with 15 nodes and an average degree of 4. In the Watts-Strogatz model, we set the rewiring probability parameter to 0.25. We generated 334 networks using each model, resulting in 1002 networks. For each network *G* generated for the eye task, we select the node with the greatest degree as the evader $v^*$ (with ties being resolved uniformly at random) following Waniek et al.^27^. We then randomly select one of the links incident to the evader as the hidden link $e^*$, and remove it from the network. Participants are then presented with an image of network *G* without the link $e^*$, and are asked to identify the hidden link.

For the shield tasks, we generate the same number of networks as for the eye task (i.e., 1002), using the same three models, and select the link to be hidden (i.e., $e^*$) following the same steps mentioned above. Following Waniek et al.^27^, we consider the effectiveness of link prediction to be the best AUC (Area under the ROC curve) score of the following algorithms: common neighbours^43^, Salton^44^, Jaccard^45^, Sørensen^46^, hub promoted^47^, hub depressed^47^, Leicht–Holme–Newman^48^, Adamic–Adar^49^, and resource allocation^50^. Out of all the links incident to either end of $e^*$, we select two links whose removal yields the greatest decrease in the effectiveness of link prediction, and two links whose removal yields the smallest decrease. Similarly, out of all the links that do not belong to *G* and are incident to either end of $e^*$, we select two whose addition yields the greatest decrease in effectiveness, and two whose addition yields the smallest decrease. As a result, we end up selecting four links that can be removed from *G*, and four that can be added to *G*. Participants are then asked to select three out of these eight possible network modifications to execute in order to hide $e^*$ from AI. Again, to discern the participants’ ability to solve the shield task, we ensure that the possible solutions include one that increases the AUC by at least 0.025, and another that decreases the AUC by at least 0.025, while also ensuring that the standard deviation of changes in AUC is at least 0.025.

### Ethics statement

The research was approved by the Institutional Review Board of the New York University Abu Dhabi (HRPP-2022-81). All research was performed in accordance with relevant guidelines and regulations. Informed consent was obtained from all participants.

### Supplementary Information

Supplementary Information.

## Data Availability

The anonymized data from the Amazon Mechanical Turk survey study is available via Figshare online repository^51^. The related code is available via Github online platform^52^.
