# Supplementary material for: Human intuition as a defense against attribute inference
Source: Sci Rep. 2023 Sep 26;13:16088. doi: 10.1038/s41598-023-43062-5 (PMC10522675; doi:10.1038/s41598-023-43062-5)
Supplement: Supplementary file 1 — Supplementary Information. [file 41598_2023_43062_MOESM1_ESM.pdf]

Supplementary materials for:
Human intuition as a defense against attribute inference

Marcin Waniek[a], Navya Suri[a,*], Abdullah Zameek[a,*],
Bedoor AlShebli[b], and Talal Rahwan[a,†]

[a]Computer Science, New York University Abu Dhabi, Abu Dhabi, UAE
[b]Social Science, New York University Abu Dhabi, Abu Dhabi, UAE
[*]Equal contributions
[†]Corresponding author: talal.rahwan@nyu.edu

A Survey questionnaires

We first briefly describe the control flow of our experiment, before listing the entire text of the questionnaire. Participating in the online survey consists of the following steps:

1. The participant is asked to accept the consent form (Section A.1).
2. The participant is asked to fill out a demographics questionnaire (Section A.2).
3. The participant is randomly assigned to one of the six treatments:
   - gender prediction eye task (Section A.3),
   - location prediction eye task (Section A.4),
   - link prediction eye task (Section A.5),
   - gender prediction shield task (Section A.6),
   - location prediction shield task (Section A.7),
   - link prediction shield task (Section A.8).

   Below, the letter X stands for a section depending on the treatment under consideration.
4. The participant is presented with a set of instructions pertaining to the task (Section A.X.1).
5. The participant is given a comprehension test about the task (Section A.X.2).
6. If the participant manages to pass the comprehension test within two attempts, they are presented with five randomly selected instances of the task (Section A.X.3); otherwise they are given a show-up fee.
7. After completing each instance, the participant is either:
   - informed whether they correctly (Section A.X.4) or incorrectly (Section A.X.5) determined the required information for the eye tasks,
   - informed about their relative effectiveness (Section A.X.4) for the shield tasks.
8. The participant is presented with a summary of payment (Section A.9).
9. The participant is asked to fill out a post-experimental questionnaire (Section A.10).
10. The participant is thanked for participation and presented with a 10-digit validation code they have to enter in the MTurk system (Section A.11).

A.1 Consent form

Welcome to this study. which is carried out by Bedoor AlShebli, Marcin Waniek, and Talal Rahwan from New York university Abu Dhabi.

Should you choose to participate in this study, you will do the following:

1. Fill out a brief questionnaire.
2. Read the instructions of the task you will be performing.

3. Answer comprehension check questions to test your understanding of the task. If you fail twice to correctly answer all questions, you will not be allowed to finish the study, but we will compensate you with $0.10 tor your time.
4. Complete the task.
5. Answer follow-up questions.

You will receive a show-up fee of $1.00 for completing the study, with the opportunity to earn more, depending on your performance. The maximum bonus you may earn in the activity is $2.5, In addition to your show-up fee.

The estimated time for the study is 12 minutes.

You are eligible to participate if you are:

1. 18 years or older
2. Live in the United States

Participation in this study is voluntary and you may leave the study at any point However, we can only pay you if you complete the study. When participating in this study we will keep track of your MTurk ID for payment and tracking. Information not containing identifiers may be used in future research or shared with other researchers without your additional consent. We do not anticipate any risks to you directly resulting from your participation in this study. There will also be no benefits to you beyond the money you earn completing the study. However, you will be assisting in the collection of valuable data about human behavior.

It you have questions about either the study or your participation, you may contact Bedoor AlShebli at bedoor@nyu.edu. For questions about your rights as a research participant, you may contact the IRB and refer to #HRPP-2019-93, New York University Abu Dhabi, +971 2628 4313 or IRBnyuad@nyu.edu. If you would like to have a copy of this document, please make a screenshot and keep it.

Important: by clicking the button below you agree to participate in the study. ONLY click this button it you intend to participate!

You can only participate in the study once. Open ONLY ONE browser window and do not attempt to duplicate your input in any way otherwise you will not get paid.

[Button with an arrow pointing right]

A.2 Demographic questionnaire

- How old are you, in years?

  [Text field accepting a number]

- What is your gender?

  [Radio button with options “Male”, “Female”, and “Other”]

- With which of the following groups do you identify? You may select more than one.

  [List of options: “White”, “Black/African-American”, “Hispanic/Latino(a)”, “Asian or Asian-American”, “American Indian or Alaska Native ”, “Middle Eastern or North African”, and “Other”]

- In which state do you currently live?

  [Drop-down list with US states]

[Button with an arrow pointing left]
[Button with an arrow pointing right]

A.3 Gender prediction eye task survey

A.3.1 Gender prediction eye task instructions

You will be presented with 5 reviews posted on the Internet. For each of them, you will be asked to determine whether the review was more likely to be posted by a female or a male*. For each review, you will receive a bonus of $0.50 if you correctly determine the required information.

Here is an example of how the user interface will look like:

[Screenshot with an example of the interface]

The button allowing you to proceed to the next page will be activated after 30 seconds. Please, take your time to read the instructions carefully as a comprehension test will be shown on the next page.

** Note that the dataset we use comes from a study in which reviewers were classified based on their biological sex.*

[Button with an arrow pointing right]

A.3.2 Gender prediction eye task comprehension test

Please answer the following questions about the rules of the activity. If you fail twice to answer all of them correctly, you will not be able to participate in the study. You can review the instructions by clicking on the "Instructions" button at the bottom of the page.

- What kind of information will be presented to you regarding each review?
  - The text of the review, a photo of the product, and the name of the reviewer
  - The text of the review and a photo of the product
  - Only the text of the review
- What is the characteristic that you will be asked to determine for each review?
  - The age of the reviewer
  - The gender of the reviewer
  - The education level of the reviewer
- How will your bonus be determined?
  - I will receive a bonus for any given review only if I correctly determine the reviewer's characteristic.
  - I will receive a bonus for every review, regardless of whether I correctly determine the reviewer's characteristic.
  - I will not receive any bonus.

[Button with "Instructions" label]
[Button with an arrow pointing right]

A.3.3 Gender prediction eye task

Task [the number of the task] of 5

Specify whether the person who posted the review below is more likely to be a female or a male.

[Text of the review]

[Radio button with options "Male", and "Female"]

[Button with an arrow pointing right]

A.3.4 Gender prediction eye task success

Well done! You have correctly determined the gender Of the reviewer. As a result, you received a bonus of $0.50

Your cumulative bonus thus far is: [Cumulative bonus value in USD]

[Button with an arrow pointing right]

A.3.5 Gender prediction eye task failure

Unfortunately, you have incorrectly determined the gender of the reviewer. The correct answer was [the correct answer]. As a result, you do not receive a bonus for this review.

Your cumulative bonus thus far is: [Cumulative bonus value in USD]

Here is a copy of the question:

[The review text from the task]

[Button with an arrow pointing right]

A.4 Location prediction eye task survey

A.4.1 Location prediction eye task instructions

You will be presented with 5 sets, each consisting of sixteen photos that were taken in the same country. For each set of photos, you will be asked to specify the country in which the photos were taken, and you will receive a bonus of $0.50 if you correctly determine the required information.

Here is an example of how the user interface will look like:

[Screenshot with an example of the interface]

The button allowing you to proceed to the next page will be activated after 30 seconds. Please, take your time to read the instructions carefully as a comprehension test will be shown on the next page.

[Button with an arrow pointing right]

A.4.2 Location prediction eye task comprehension test

Please answer the following questions about the rules of the activity. If you fail twice to answer all of them correctly, you will not be able to participate in the study. You can review the instructions by clicking on the "Instructions" button at the bottom of the page.

- How many photos will each set consist of?
  - 1 photo
  - 8 photos
  - 16 photos

- What is the attribute that you will be asked to determine for each set of photos?
  - The year in which the photos were taken
  - The country in which all the photos were taken
  - Whether the photos were taken by the same person
- How will your bonus be determined?
  - I will receive a bonus for any given set of photos only if I correctly determine the photos' attribute.
  - I will receive a bonus, regardless of my performance.
  - I will not receive any bonus.

[Button with "Instructions" label]
[Button with an arrow pointing right]

A.4.3 Location prediction eye task

Task [the number of the task] of 5
In which country do you think the following photos were taken?
[A set of 16 photographs taken in the same country]
From [Drop-down list with countries]
[Button with an arrow pointing right]

A.4.4 Location prediction eye task success

Well done! You have correctly determined the country in which the photos were taken. As a result, you received a bonus of $0.50
Your cumulative bonus thus far is: [Cumulative bonus value in USD]
[Button with an arrow pointing right]

A.4.5 Location prediction eye task failure

Unfortunately, you have incorrectly determined the country in which the photos were taken. The correct answer was [the correct answer]. As a result, you do not receive a bonus for this task.
Your cumulative bonus thus far is: [Cumulative bonus value in USD]
Here is a copy of the question:
[Picture of the set of photographs from the task]
[Button with an arrow pointing right]

A.5 Link prediction eye task survey

A.5.1 Link prediction eye task instructions

You will be presented with 5 social networks, each consisting of nodes (which represent people) and links (which represent friendships). In each network, two individuals know each other (meaning that there is supposed to be a friendship link between them), but they pretend that they do not know each other (meaning that the link between them is not part of the network). You will be

asked to guess who these two individuals are, based on the structure of the social network. For each network, you will receive a bonus of $0.50 if you correctly determine the required information.

Here is an example of how the user interface will look like:

[Screenshot with an example of the interface]

The button allowing you to proceed to the next page will be activated after 30 seconds. Please, take your time to read the instructions carefully as a comprehension test will be shown on the next page.

[Button with an arrow pointing right]

A.5.2 Link prediction eye task comprehension test

Please answer the following questions about the rules of the activity. If you fail twice to answer all of them correctly, you will not be able to participate in the study. You can review the instructions by clicking on the “Instructions” button at the bottom of the page.

- What does each network represent?
  - Streets within a city
  - Connections between airports
  - Friendships between people
- What is the information you will be required to determine for each network?
  - The number of links it has
  - Which two nodes have an undisclosed link between them
  - Which node is the most important
- How will your bonus be determined?
  - I will receive a bonus for any given network only if I correctly determine the required information.
  - I will receive a bonus for every network, regardless of whether I correctly determine the required information.
  - I will not receive any bonus.

[Button with “Instructions” label]

[Button with an arrow pointing right]

A.5.3 Link prediction eye task

Task [the number of the task] of 5

In this social network, two individuals know each other (meaning that there is supposed to be a friendship link between them), but they pretend that they do not know each other (meaning that the link between them is not part of the network). Based on the network structure, who are these two individuals?

[Picture of a network]

From [Drop-down list with network nodes]

To [Drop-down list with network nodes]

[Button with an arrow pointing right]

A.5.4 Link prediction eye task success

Well done! You have correctly identified the two nodes that have an undisclosed link between them. As a result, you received a bonus of $0.50

Your cumulative bonus thus far is: [Cumulative bonus value in USD]

[Button with an arrow pointing right]

A.5.5 Link prediction eye task failure

Unfortunately, you have incorrectly determined the two nodes that have an undisclosed link between them. The correct answer was [the correct answer]. As a result, you do not receive a bonus for this task.

Your cumulative bonus thus far is: [Cumulative bonus value in USD]

Here is a copy of the question:

[Picture of the network from the task]

[Button with an arrow pointing right]

A.6 Gender prediction shield task survey

A.6.1 Gender prediction shield task instructions

You will be presented with 5 reviews posted on the internet. For each review, you will be informed about the gender of the person who wrote the review. Moreover, you will be asked to replace three words with their synonyms, in order to make it harder for an AI algorithm to correctly guess that person's gender*. To this end, you will be presented with eight word substitutions to choose from. For example, the substitution "husband" → "hubby" means that the word "husband" in the review will be replaced with the word "hubby". The harder you make it for an AI algorithm to correctly guess the reviewer's gender, the greater your bonus. For each review, the maximum bonus you may receive is $0.50.

Here is what the user interface will look like. As you can see, it displays a sample review, followed by eight suggested substitutions:

[Screenshot with an example of the interface]

The button allowing you to proceed to the next page will be activated after 30 seconds. Please, take your time to read the instructions carefully as a comprehension test will be shown on the next page.

** Note that the dataset we use comes from a study in which reviewers were classified based on their biological sex.*

[Button with an arrow pointing right]

A.6.2 Gender prediction shield task comprehension test

Please answer the following questions about the rules of the activity. If you fail twice to answer all of them correctly, you will not be able to participate in the study. You can review the instructions by clicking on the "Instructions" button at the bottom of the page.

- Your task is to make it harder for an AI algorithm to guess certain information. What is this information?

- The reviewer's age
  - Whether review written by a human or by an AI
  - The reviewer's gender

- How will you perform this task?
  - By adding two sentences to the review
  - By replacing three words in the review
  - By writing a completely new review

- How will your bonus be determined?
  - The harder I make it for an AI algorithm to correctly guess the information, the greater my bonus.
  - I will always receive a bonus, regardless of my performance.
  - I will not receive any bonus.

[Button with "Instructions" label]
[Button with an arrow pointing right]

A.6.3 Gender prediction shield task

Task [the number of the task] of 5
[Text of the review]

The person Who wrote this review is [the gender of the author]. Which three substitutions do you propose in order to make it harder for an AI algorithm to correctly guess that person's gender? (Drag your choices into the box)

Possible choices [List of eight possible modifications]
Your choices [List to which three modifications can be dragged]
[Button with an arrow pointing right]

A.6.4 Gender prediction shield task feedback

The effectiveness of your solution was [relative effectiveness of the participants answer] compared to the best solution. The best solution was [the best solution]. As a result, you received a bonus of [the bonus increment in USD]

Your cumulative bonus thus far is: [Cumulative bonus value in USD]
[Button with an arrow pointing right]

A.7 Location prediction shield task survey

A.7.1 Location prediction shield task instructions

A recent study has shown that when a person posts photos on the internet, an AI algorithm may be able to correctly guess the country in which these photos were taken.

You will be presented with 5 sets, each consisting of sixteen photos that were taken in the same country. For each set, you will be informed about the country in which the photos were taken. Moreover, you will be asked to remove three photos from the set, in order to make it harder for an AI algorithm to correctly guess the country. To this end, you will be presented with eight photos to

choose from. The harder you make it for an AI algorithm to correctly guess the country in which the photos were taken, the greater your bonus. For each set of photos, the maximum bonus you may receive is $0.50.

Here is what the user interface will look like. As you can see, it displays a sample set of sixteen photos; the photos you can choose from are highlighted by a red border, along with a letter associated with each photo:

[Screenshot with an example of the interface]

The button allowing you to proceed to the next page will be activated after 30 seconds. Please, take your time to read the instructions carefully as a comprehension test will be shown on the next page.

[Button with an arrow pointing right]

A.7.2 Location prediction shield task comprehension test

Please answer the following questions about the rules of the activity. If you fail twice to answer all of them correctly, you will not be able to participate in the study. You can review the instructions by clicking on the "Instructions" button at the bottom of the page.

- Your task is to make it harder for an AI algorithm to guess certain information. What is this information?
  - The year in which the photos were taken
  - The country in which the photos were taken
  - Whether the photos were taken by a human or by an AI
- How will you perform this task?
  - By adding three photos to the set
  - By removing three photos from the set
  - By adding a photo to and removing a photo from the set
- How will your bonus be determined?
  - The harder I make it for an AI algorithm to correctly guess the information, the greater my bonus.
  - I will always receive a bonus, regardless of my performance.
  - I will not receive any bonus.

[Button with "Instructions" label]

[Button with an arrow pointing right]

A.7.3 Location prediction shield task

Task [the number of the task] of 5

The country in which these photos were taken is [the country name]. Out of the eight photos highlighted in red, which three do you propose we remove in order to make harder for an AI algorithm to correctly guess the country? (Drag your choices into the box)

[A set of 16 photographs taken in the same country]

Possible choices [List of eight possible modifications]

Your choices [List to which three modifications can be dragged]

[Button with an arrow pointing right]

A.7.4 Location prediction shield task feedback

The effectiveness of your solution was [relative effectiveness of the participants answer] compared to the best solution. The best solution was [the best solution]. As a result, you received a bonus of [the bonus increment in USD]

Your cumulative bonus thus far is: [Cumulative bonus value in USD]

[Button with an arrow pointing right]

A.8 Link prediction shield task survey

A.8.1 Link prediction shield task instructions

A social network consists of nodes (which represent people) and links (which represent friendships). In such a network, it is possible that two individuals know each other (meaning that there is supposed to be a friendship link between them), but they pretend that they do not know each other (meaning that the link between them is not part of the network). A recent study has shown that, in such cases, an AI algorithm may be able to correctly guess who these two individuals are.

You will be presented with 5 social networks. For each network, you will be informed about the identity of the individuals who pretend to not know each other. Moreover, you will be asked to introduce three modifications to the network, in order to make it harder for the Al algorithm to correctly guess who these two individuals are. To this end, you will be presented with eight possible modifications to choose from (these consist of four links that can be added to the network, and four links that can be removed). The harder you make it for an AI algorithm to correctly guess the individuals who pretend to not know each other, the greater your bonus. For each network, the maximum bonus you may receive is $0.50.

Here is what the user interface will look like. As you can see, it displays a sample network. The yellow (dashed) link connects the two individuals who pretend to not know each other (you can see this link, but the Al algorithm cannot). The four blue (dotted) links can be added. The red (bold) links can be removed:

[Screenshot with an example of the interface]

The button allowing you to proceed to the next page will be activated after 30 seconds. Please, take your time to read the instructions carefully as a comprehension test will be shown on the next page.

[Button with an arrow pointing right]

A.8.2 Link prediction shield task comprehension test

Please answer the following questions about the rules of the activity. If you fail twice to answer all of them correctly, you will not be able to participate in the study. You can review the instructions by clicking on the "Instructions" button at the bottom of the page.

- Your task is to make it harder for an AI algorithm to guess certain information. What is this information?
  - The number of links the network has

- Which node is the most important one in the network
- Which two individuals have an undisclosed link between them

- How will you perform this task?
  - By adding five links
  - By adding or removing three links
  - By removing four link

- How will your bonus be determined?
  - The harder I make it for an AI algorithm to correctly guess the information, the greater my bonus.
  - I will always receive a bonus, regardless of my performance.
  - I will not receive any bonus.

[Button with “Instructions” label]
[Button with an arrow pointing right]

A.8.3 Link prediction shield task

Task [the number of the task] of 5

The yellow (dashed) link [the label of the hidden link] connects the two individuals who pretend to not know each other. The picture highlights eight possible modifications: four blue (dotted) links that can be added to the network, and four red (bold) links that can be removed from the network. Out of these eight modifications, which three do you propose in order to make it harder for an AI algorithm to correctly guess who these two individuals are?

[Picture of a network]

Possible choices [List of eight possible modifications]

Your choices [List to which three modifications can be dragged]

[Button with an arrow pointing right]

A.8.4 Link prediction shield task feedback

The effectiveness of your solution was [relative effectiveness of the participants answer] compared to the best solution. The best solution was [the best solution]. As a result, you received a bonus of [the bonus increment in USD]

Your cumulative bonus thus far is: [Cumulative bonus value in USD]

[Button with an arrow pointing right]

A.9 Payment summary

Thank you for your participation!

Your payment (including any bonuses you may have earned) is: [Total payment in USD].

You will receive your payment once you finish answering a few follow-up questions. Click ”Continue” to answer a few questions about yourself and the task.

[Button with “Continue” label]

A.10 Post-experimental questionnaire

- What is your attitude towards Artificial Intelligence (Al)?

  [Radio button with options "Extremely Positive", "Positive", "Slightly Positive", "Neutral", "Slightly Negative", "Negative", and "Extremely Negative"]

- What is the highest Of education you have completed?

  [Radio button with options "Less than high school", "High school or equivalent (e.g. GED)", "Some college", "2-year degree (Associate's)", "4-year degree (Bachelor's)", "Graduate or professional degree"]

- What was your total family income from all sources in 2021, before taxes?

  [Radio button with options "Less than $10,000", "$10,000 to $19,999", "$20,000 to $29,999", "$30,000 to $39,999", "$40,000 to $49,999", "$50,000 to $59,999", "$60,000 to $69,999", "$70,000 to $79,999", "$80,000 to $89,999", "$90,000 to $99,999", "$100,000 to $150,000", "More than $150,000"]

- Please, tell us your 5-digit zip-code. This information will only be used for statistical purposes.

  [Text field accepting five digit code]

- Was there anything noteworthy about the study?

  [Text field]

- How would you rate your overall experience completing this study?

  [Radio button with options "Extremely Positive", "Positive", "Slightly Positive", "Neutral", "Slightly Negative", "Negative", and "Extremely Negative"]

[Button with an arrow pointing left]
[Button with "Continue" label]

A.11 End screen

Thank you for your participation!

Your payment (including any bonuses you may have earned) is: [Total payment in USD]
Your validation code is: [10-digit validation code]

B Supplementary tables

Task	Attribute	Participants	t-statistic	p-value
Eye	Gender	216	9.719	7.048×10^{-22}
	Location	340	10.074	1.530×10^{-23}
	Link	200	3.708	2.152×10^{-4}
Shield	Gender	150	21.779	1.581×10^{-91}
	Location	149	54.786	5.422×10^{-313}
	Link	110	25.216	8.290×10^{-109}

Table S1: **Statistical values corresponding to our experiment.** The table specifies the number of participants who performed each task, as well as the results of the Welch's t-test (the t-statistic and the p-value) comparing the performance of participants vs. AI.
